# A simple *in vitro* method to measure autophosphorylation of protein kinases

**DOI:** 10.1186/1746-4811-9-22

**Published:** 2013-06-26

**Authors:** Isaiah Taylor, Kati Seitz, Stefan Bennewitz, John C Walker

**Affiliations:** 1 Division of Biological Sciences, University of Missouri, Columbia, MO 65211, USA; 2 Interdisciplinary Plant Group, University of Missouri, Columbia, MO 65211, USA; 3 Present address: Department of Cell and Metabolic Biology, Leibniz Institute of Plant Biochemistry, Halle (Saale), Germany

**Keywords:** Autophosphorylation, *In vitro* protein kinase assay, Protein kinase, Receptor-like protein kinase, RLK

## Abstract

Receptor-like protein kinases (RLKs) are a large and important group of plant proteins involved in numerous aspects of development and stress response. Within this family, homo-oligermization of receptors followed by autophosphorylation of the intracellular protein kinase domain appears to be a widespread mechanism to regulate protein kinase activity. *In vitro* studies of several RLKs have identified autophosphorylation sites involved in regulation of catalytic activity and signaling *in vivo*. Recent work has established that multiple RLKs are biochemically active when expressed in *E. coli* and readily autophosphorylate prior to purification or subsequent manipulation. This observation has led us to develop a simplified method for assaying RLK phosphorylation status as an indirect measure of intrinsic autophosphorylation activity. The method involves expressing a recombinant RLK protein kinase domain in *E. coli*, followed by SDS-PAGE of boiled cell lysate, and sequential staining with the phosphoprotein stain Pro-Q Diamond and a colloidal Coomassie total protein stain. We show this method can be used to measure and quantify *in vitro* autophosphorylation levels of recombinant wildtype and mutant versions of the Arabidopsis RLK HAESA, as well as to detect transphosphorylation activity of recombinant HAESA against a protein kinase inactive version of itself. Our method has several advantages over traditional protein kinase assays. It does not require protein purification, transfer, blotting, or radioactive reagents. It allows for rapid and quantitative assessment of autophosphorylation levels and should have general utility in the study of any autophosphorylating protein kinase expressed in *E. coli*.

## Background

Autophosphorylation is a common activation mechanism of protein kinases. Receptor-like protein kinases (RLK)s, the largest group of protein kinases in the Viridiplantae, have been shown to possess widespread autophosphorylation activity *in vitro*[1,2], and *in vivo* functional characterization has demonstrated homo- and hetero-oligomerization of multiple RLKs [3-5]. These observations support the notion that autophosphorylation of receptor molecules, followed by transphosphorylation by co-receptors, is a conserved early event in RLK-mediated signal transduction. Therefore, the study of autophosphorylation is an important area of RLK research with implications in diverse areas of plant biology.

Initial studies of RLK autophosphorylation were carried out using purified recombinant protein kinase expressed in *E. coli*, followed by *in vitro* kinase assays using radiolabelled ATP [1]. This method of phosphorylation detection has largely been superseded by those utilizing phosphothreonine/serine/tyrosine antibodies, and those utilizing the general phospho-amino acid stain Pro-Q Diamond [6], eliminating the drawbacks associated with radioactive compounds. In a further advance, it has recently been observed that many autophosphorylating protein kinases are highly phosphorylated when expressed in *E. coli* prior to any additional *in vitro* kinase reactions, including the Arabidopsis RLKs BRI1 [7,8] and HAE [described herein].

Innovative work focusing on BRI1 has taken advantage of this phenomenon to develop what have been termed *in situ* kinase assays [8]. This work has shown that detection of autophosphorylation of BRI1 and related kinases can be accomplished directly following protein production in *E. coli*. Work by Oh *et al.* has also shown the utility of assessing transphosphorylation activity of recombinant kinases against endogenous *E. coli* proteins, as well as a technique for coexpressing BRI1 with putative negative regulators of protein kinase activity [8,9]. This work demonstrates the versatility of *E. coli* not only as a means to produce recombinant protein kinases, but also as the reaction vessel in which to perform autophosphorylation assays.

Here, we describe an advancement of this *in situ* protein kinase approach using the Arabidopsis RLK HAESA (HAE) as a model. Our method is a simple quantitative assay of protein kinase autophosphorylation levels using boiled cell lysates from *E. coli* expressing the recombinant protein kinase, followed by SDS-PAGE and sequential staining with the phosphoamino acid stain Pro-Q Diamond and total protein staining with Coomassie Blue Silver [10]. This protocol requires no protein purification or membrane transfer. It is feasible because signal of the recombinant protein kinase dominates after staining with Pro-Q Diamond. Surprisingly, there is essentially no background staining from endogenous *E. coli* phosphoproteins. Coupling the phosphoprotein staining with a total protein stain allows for normalization of protein expression level, and quantitative assessment of autophosphorylation. We also demonstrate the ability to detect *in situ* transphosphorylation by recombinant HAE against a protein kinase inactive version of itself. This approach should have general utility in the study autophosphorlyation and transphosphorylation of a large number of auto-activating kinases. The benefits of this approach are its speed, simplicity, and consistency.

## Results and discussion

### Autophosphorylation of recombinant HAESA is readily detectable from *E. coli* boiled cell lysate using Pro-Q Diamond

**Figure 1:**
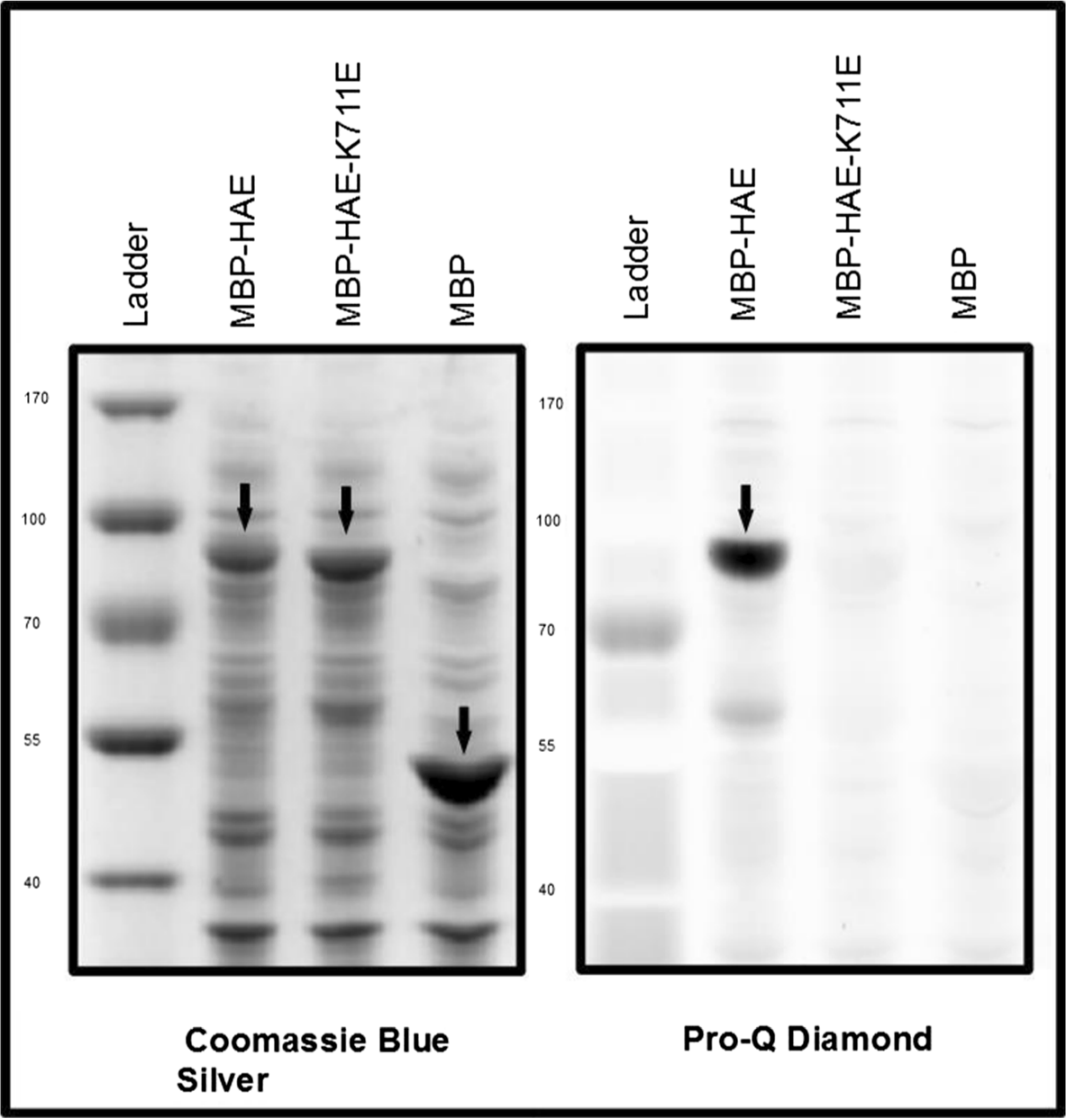
**Detection of recombinant HAE in *E. coli *cell lysates by Pro-Q Diamond phosphoprotein stain.** Left: Total protein stain showing gel shifted MBP-HAE run alongside MBP-HAE-K711E and MBP parental vector negative control. Right: phosphoprotein stain showing heavy autophosphorylation of MBP-HAE and near complete absence of signal in MBP-HAE-K711E and MBP parental vector lanes. Arrows point to bands identified by lane labels.

The initial observation that led us to develop this protocol was that following the induction of expression of a Maltose Binding Protein-HAE protein kinase catalytic domain recombinant fusion protein (MBP-HAE), followed by SDS-PAGE of a boiled cell lysate, and staining of the gel with Pro-Q Diamond phospho-amino acid stain, we could observe an extremely strongly stained band near the predicted size of the MBP-HAE fusion protein at 80.5 KDa [Figure 1]. This signal is dependent on HAE protein kinase activity, as a kinase inactive mutant of HAE with a substitution of glutamic acid for the conserved catalytic lysine at position K711 (MBP-HAE-K711E) is undetected by Pro-Q Diamond, as is MBP expressed alone from the parental vector as a negative control. We attribute minor differences in apparent size to the gel shift resulting from multiple phosphorylation sites on the wildtype protein.

In the MBP-HAE lane, we consistently observe a smaller band that stains with Pro-Q Diamond and Coomassie, as well as a slight smear below MBP-HAE. We believe these are degradation products of HAE, because their staining with Pro-Q Diamond is dependent on HAE kinase activity, and because they are absent from the MBP parental vector control lane. A bench protocol for this experiment has been included as Additional file 1.

### Validation of quantification method

The above approach can be used to qualitatively assess autophosphorylation levels. We were also interested in developing a technique that could be used to measure autophosphorylation quantitatively. To do so, we used the following equation to calculate a background-adjusted, protein abundance-normalized signal ratio for each band of interest:

$$\begin{array}{l} \mathrm{Signal}\;\mathrm{Ratio}=\left(\mathrm{ProQ}\;\mathrm{signal}–\mathrm{ProQ}\;\mathrm{Background}\right)/\\ \;\left(\mathrm{Coomassie}\;\mathrm{signal}–\mathrm{Coomassie}\;\mathrm{Background}\right) \end{array}$$

Based on analysis of the linear range of detection for both Pro-Q Diamond and Coomassie Blue Silver of MBP-HAE [See Additional file 2], it was empirically determined that under our expression conditions, protein derived from between 4–8 μL of liquid culture yielded an amount of protein within the maximal linear range of detection for both stains. In subsequent experiments, we have analyzed 5 μL of sample to fall near the midpoint of this range.

**Figure 2:**
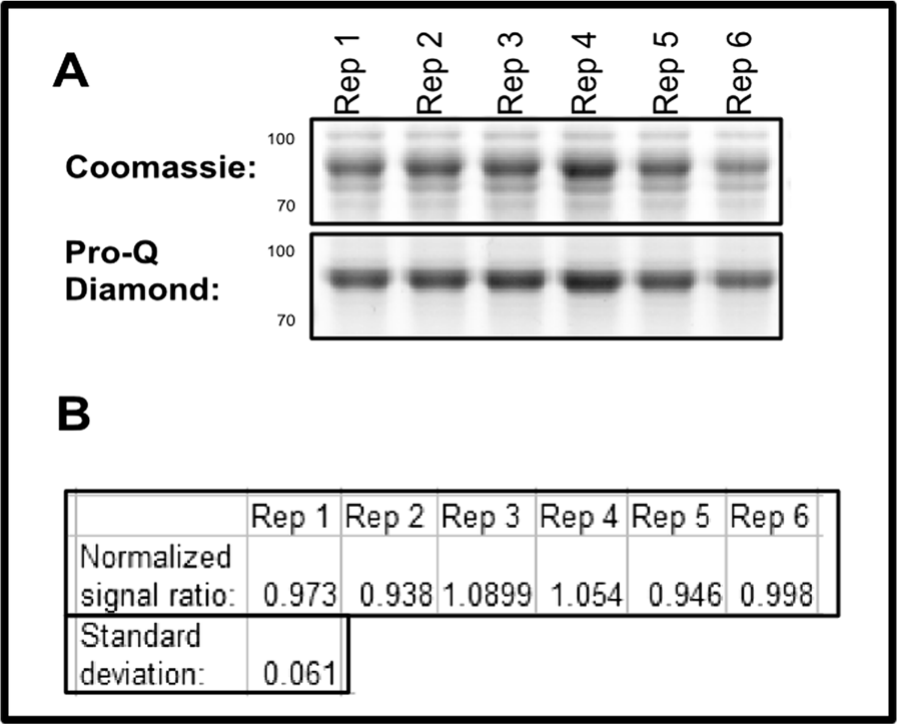
**Signal ratio of Pro-Q Diamond to total protein is stable across biological replicates. A**: Total protein and phosphoprotein stains of gel containing 6 biological replicate culture of MBP-HAE. **B**: Background corrected signal ratio normalized to the average for 6 biological replicates with standard deviation.

To demonstrate the reproducibility of this approach, 6 biological replicates of MBP-HAE were grown and protein expression induced. 5 μL for each biological replicate was run on a gel [Figure 2A]. Band intensity of the 6 MBP-HAE biological replicates was estimated for both Pro-Q Diamond and Coomassie Blue Silver, and the background signal from an MBP parental vector control lane was subtracted to yield background corrected signal estimates. Next, the Pro-Q Diamond signal estimate was divided by the Coomassie signal estimate for each sample to yield the background-adjusted signal ratio [Figure 2B].

**Figure 3:**
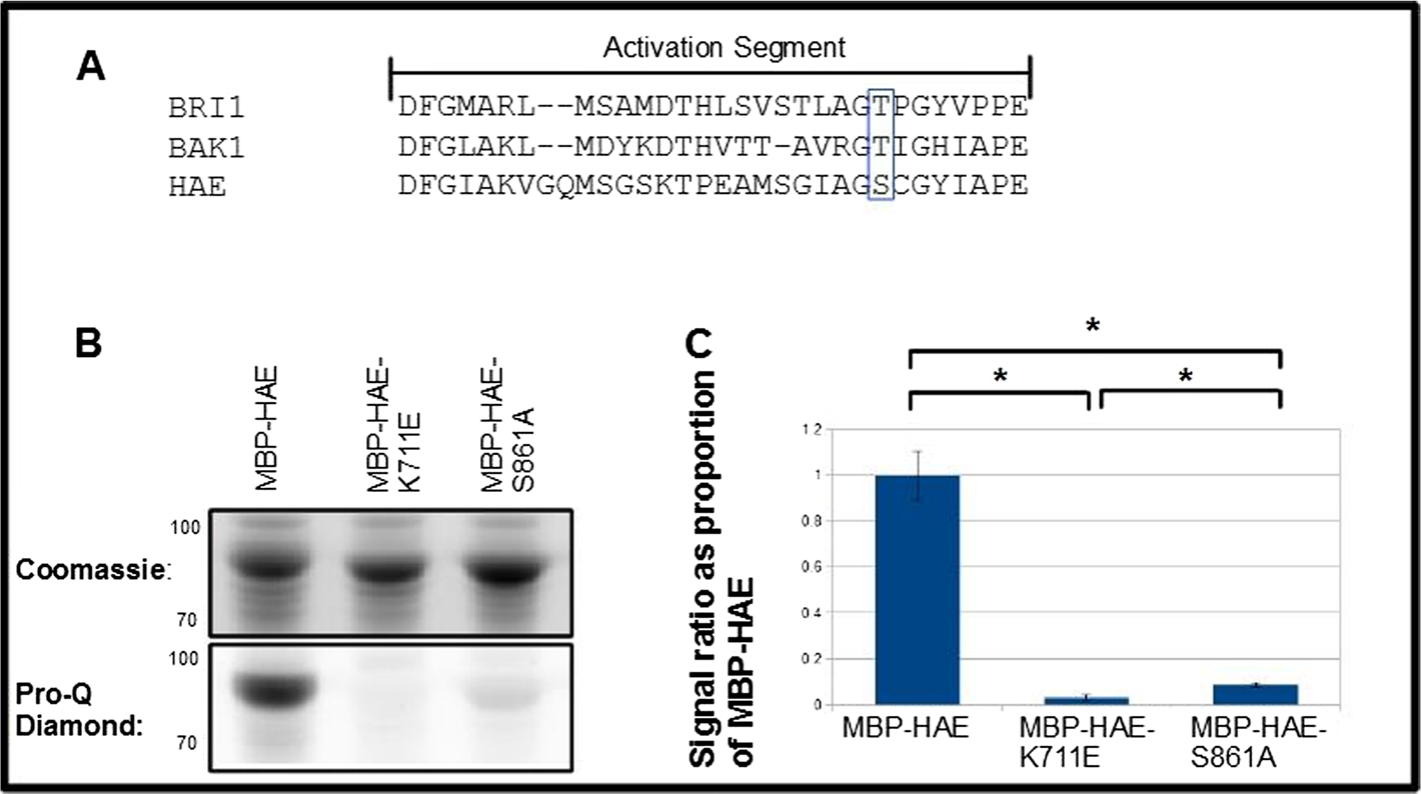
**Mutational analysis identifies a conserved serine residue important for HAE protein kinase function. A**: Alignment of activation segment of 3 Arabidopsis RLKs. Blue box contains BRI1-T1049, BAK1-T455, and HAE-S861. **B**: Total protein and phosphoprotein stains of gel containing MBP-HAE, MBP-HAE-K711E, and MBP-HAE-S861A. **C**: Average wildtype-normalized signal for MBP-HAE, MBP-HAE-K711E, and MBP-HAE-S861A across three sets of biological replicates run on a single gel. * indicates significance at p-value < .01, Student's *t*-test. Error bars represent standard deviation of 3 biological replicates.

Despite variation in protein abundance of +/− 15% of the average, the variation of the signal ratio for the replicates is quite low, with the standard deviation calculated to be 6% of the average ratio [Figure 3B]. This indicates that the normalized autophosphorylation ratios measured with this assay exhibits sample to sample stability. A diagram of this workflow has been included as Additional file 3.

### Demonstration of utility in mutational analysis

We anticipate the primary application of this technique to be in mutational analysis of autophosphorylating protein kinases. To demonstrate its utility, we performed a literature search and homology mapping to identify a conserved serine residue (S861) in the activation segment of HAE [Figure 3A]. This residue aligns to a serine or threonine in most serine/threonine protein kinases [11], and mutational analysis of several RLKs, including BRI1 and BAK1, has demonstrated its importance in protein kinase activity and signaling *in vivo*[5,6].

Consistent with previous findings with BRI1 and BAK1 [5,6], an alanine substitution mutant of HAE, HAE-S861A, shows nearly complete loss of staining with Pro-Q Diamond [Figure 3B], indicating that this serine plays an essential role in HAE protein kinase activity. Interestingly, there remains a statistically significantly higher level of Pro-Q Diamond detection of HAE-S861A compared to HAE-K711E, indicating that the HAE-S861A mutant is a severely impaired but not fully inactive kinase.

### Detection of transphosphorylation activity

**Figure 4:**
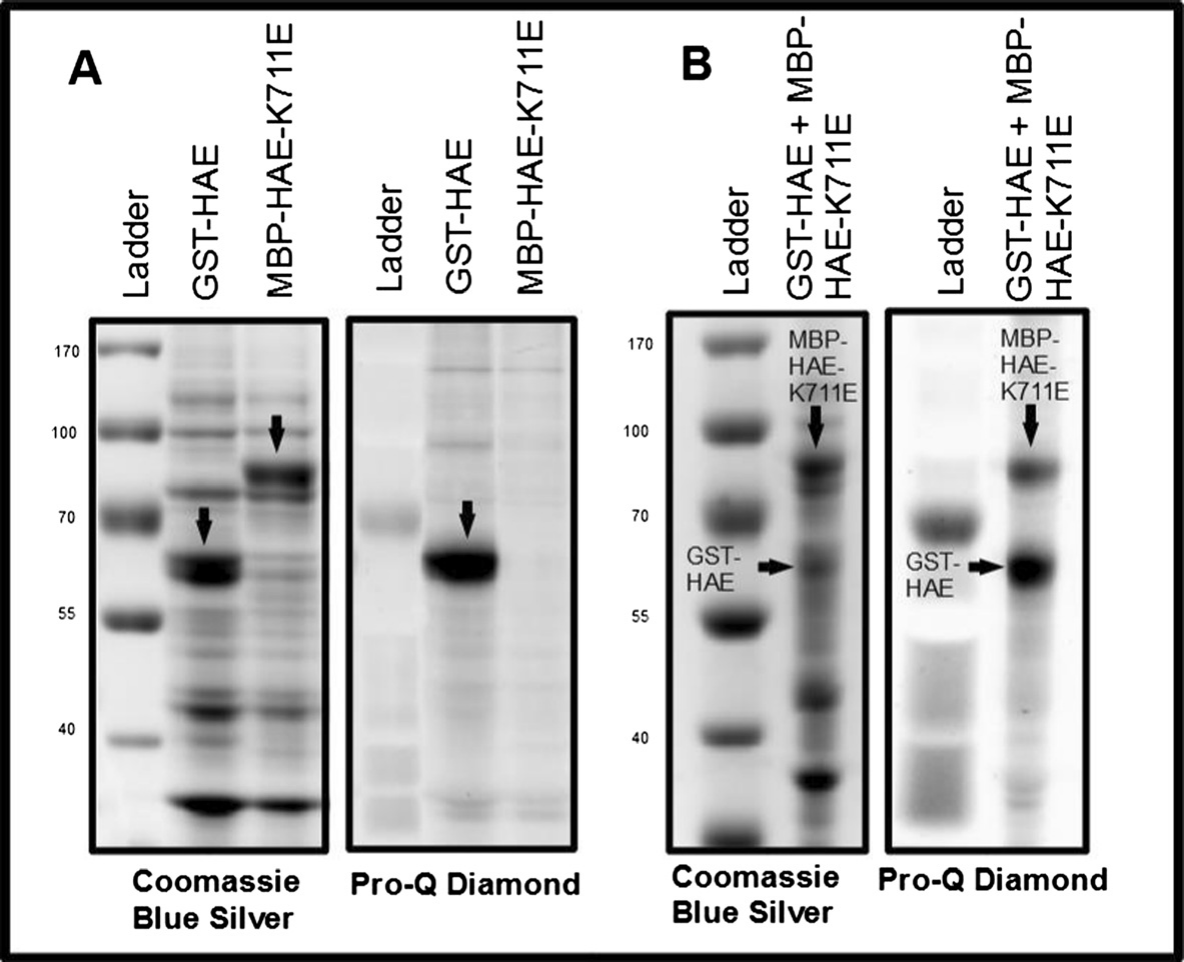
**Transphosphorylation of an inactive version of HAE by active GST-HAE. A**: Total protein and phosphoprotein stains of GST-HAE and MBP-HAE-K711E. **B**: Total protein and phosphoprotein stains of coexpressed GST-HAE and MBP-HAE-K711E. Arrows point to bands identified by lane labels.

We also attempted to detect autophosphorylation of a predicted 65.6 KDa Glutathione S-tranferase-HAE fusion protein (GST-HAE) in an identical manner as above, run alongside an MBP-HAE-K711E negative control [Figure 4A]. This result demonstrates the general application of this technique to the study of protein kinases fused to multiple tags. It should be noted that our empirically optimized induction conditions for the MBP-HAE fusion protein and GST-HAE fusion protein differed in both temperature and duration (25 degrees C for 4 hours and 37 degrees C for 2 hours, respectively).

Kinetic analysis of autophosphorylation of HAE has indicated that the process is likely to occur via both an inter- and intra-molecular mechanism [1]. Based on the size difference of the GST-HAE and MBP-HAE fusion proteins, we reasoned that upon cotransformation into *E. coli* and coexpression of these two constructs, we may be able to detect strict trans-autophosphorylation of MBP-HAE-K711E by GST-HAE using this assay. This is in fact what was observed [Figure 4B]. In this experiment, after swapping resistance of the pMAL-HAE-K711E vector from ampicillin to spectinomycin, cotransforming *E. coli* with both pMAL-HAE-K711E-SpecR and pGEX-GST-HAE-AmpR, and testing several coexpression conditions, we determined that the highest trans-autophosphorylation signal was observed when expressing these constructs in conditions optimized for MBP-HAE (ie, 25 C for 4 hours). Despite clearly lower GST-HAE protein levels, both the region of the gel containing GST-HAE and MBP-HAE-K711E display significant signal when stained with Pro-Q Diamond. We attribute higher background staining to the increased sensitivity of the Biorad ChemiDoc autoexposure due to relatively lower overall Pro-Q Diamond staining.

While further optimization of this technique for a given protein kinase of interest will no doubt lead to more robust protein expression, this work demonstrates that autophosphorylation in trans is readily detectable using this assay. We expect that coexpression from a single plasmid, rather than from two cotransformed plasmids, will significantly improve phosphorylation detection. In addition to testing intermolecular autophosphorylation, it suggests that coexpression of autoactivating protein kinases with downstream substrates may provide a simple system for studying protein kinase/substrate interactions.

## Conclusions

We have described a simplified assay for measuring autophosphorylation of recombinant protein kinases. While this approach is likely suitable to the study of many autoactivating protein kinases, it should be noted that a failure to detect autophosphorylation using this approach is not sufficient evidence to conclude a protein kinase lacks autophosphorylation activity. Effects of the heterologous cellular environment may prevent autophosphorylation of a given recombinant protein kinase. Additionally, this method of direct assessment of autophosphorylation depends on high levels of recombinant protein expression, which may not be attainable for every protein kinase. In such cases, affinity purification may be required to increase sample purity.

Nonetheless, in cases where high protein expression is achievable and autophosphorylation is detectable, the simplicity and reproducibility of this assay make it an excellent tool for investigating structure/function relationships by mutational analysis. We have further shown that its use extends to the study of trans-autophosphorylation, and in principle could be extended to the study of coexpressed protein kinase/substrate pairs. Taken together, these results demonstrate the broad utility of this method.

## Methods

### Expression constructs

Expression constructs have been previously described [1], except for pMAL-HAE-S861A, which was created by performing site directed mutagenesis on the previously described pMAL-HAE vector. pMAL-HAE-K711E-SpecR was created by blunt end ligation of an inverse PCR product deleting the ampicillin resistance gene from pMAL-HAE-K711E and a polynucleotide kinase treated PCR amplicon of the spectinomycin resistance gene from pCR8. The ligation was carried out at overnight at 4°C. The product was transformed into *E. coli* strain Dh5α for single colony isolation, before subsequent transformation into *E. coli* strain BL21 containing pGEX-HAE for MBP-HAE-K711E/GST-HAE coexpression experiments. Constructs were sequence verified. A list of primers has been included as Additional file 4.

### Protein expression and sample preparation

All bacterial cultures were grown in a temperature controlled platform incubator shaking at 300 RPM. *E. coli* strain BL21 harboring expression plasmids were grown overnight for >20 hours in LB containing appropriate antibiotics at 37°C to saturation. 35 μL of the overnight cultures were inoculated into 1 mL of fresh LB containing appropriate antibiotics. These were allowed to grow for approximately 3 hours at 37°C until reaching OD_600_ of 0.6-0.8, at which point IPTG was added to yield a final concentration of 0.1 mM. The cultures were then grown at 25°C for an additional 4 hours for the MBP fusion expression and GST/MBP fusion coexpression experiments, or 37°C for 2 hours for GST fusion expression, at which point cells from 100 μL were harvested by centrifugation. The supernatant was removed and the cells were either flash frozen in liquid nitrogen for later analysis or 100 μL of SDS sample buffer (62.5 mM Tris-Cl, pH 6.8, 2%(w/v) SDS, 10%(v/v) glycerol, 1% ß-mercaptoethanol, 0.005% bromophenol blue) was directly added to the cell pellet and the sample was resuspended by pipetting, boiled for 3 minutes, spun at max speed in a microcentrifuge to pellet insoluble debris, and the resulting supernatant was used in the Pro-Q Diamond assay.

### Pro-Q diamond and coomassie blue silver staining

For Pro-Q Diamond staining [Life Technologies, Carlsbad, California], we utilized the protocol developed by Agrawal and Thelen [12]. In brief, SDS-PAGE was performed on 5 μL of the boiled lysate from above. Next, the gel was incubated once in fixation solution for 30 minutes (50% methanol, 10% acetic acid), drained, incubated again in fixation solution overnight, washed twice in deionized water for 30 minutes, immersed in 50 mL 1/3rd strength Pro-Q Diamond solution (Pro-Q Diamond solution from manufacturer diluted 1:3 with deionized water) for 2 hours in the dark, washed four times with destain solution for 30 minutes each time (20% acetonitrile, 50 mM sodium acetate pH 4.0), before being washed in deionized water twice for 5 minutes, and imaged using the Pro-Q Diamond protocol on a BioRad ChemiDoc MP Imager with default settings. Data was saved for later analysis as described below, and the gels were immersed in 50 mL Blue Silver Coomassie stain (0.12% Coomassie Blue G-250, 10% ammonium sulfate, 10% phosphoric acid, and 20% methanol) for at least one hour, and destained for one hour in destaining solution (10% Methanol/10% Acetic Acid), followed by washing twice in deionized water for 5 minutes and imaging using the Coomassie staining protocol on a BioRad GelDoc EZ Imager with default settings.

### Band quantity estimate

Estimates of band staining intensity from both Pro-Q Diamond images and Coomassie staining images were made using BioRad Image Lab software. Lanes and bands were auto-detected and manually size-adjusted to size to ensure full band inclusion and equivalent thickness across all lanes. For the MBP parental background staining estimation, an equally sized area was manually defined in the region were MBP-HAE resided when run on the same gel. One wildtype band per gel was selected as the normalizing reference, whose default value is 1.

Background signal correction was performed by subtracting the Pro-Q Diamond and Coomassie background value derived from the MBP lane from the respective sample values. Signal ratios were calculated by dividing the background corrected Pro-Q Diamond value by the background corrected Coomassie Blue Silver value. These ratios were then normalized to the average ratio (for the biological replicate analysis) or to the average of the three wildtype replicates for the S861A analysis. See Supplemental Figure 2 for diagrammatic description of this process. Significance for the MBP-HAE-S861A experiment was calculated using Student's *t*-test.

## Abbreviations

RLK: Receptor-like protein kinase; HAE: HAESA; MBP: Maltose binding protein; GST: Glutathione S-Transferase; BRI1: BRASSINOSTEROID-INSENSITIVE1; BAK1: BRI1-ASSOCIATED RECEPTOR KINASE 1.

## Competing interests

The authors declare that they have no competing interests.

## Authors’ contributions

SB and JW conceived of the method. SB and IT designed and performed experiments. IT and KS optimized experimental conditions. IT drafted the manuscript. SB and KS provided feedback on the manuscript. JW provided comments and guidance at all stages. All authors read and approved the final manuscript.

## Supplementary Material

Additional file 1 Bench protocol for MBP-HAE expression and staining. Click here for file

Additional file 2 Linear range analysis of Coomassie Blue Silver and Pro-Q Diamond. Click here for file

Additional file 3 Diagram of quantification workflow. Click here for file

Additional file 4 Primer sequences. Click here for file
