# Supplementary material for: A simple in vitro method to measure autophosphorylation of protein kinases
Source: Plant Methods. 2013 Jun 26;9:22. doi: 10.1186/1746-4811-9-22 (PMC3702502; doi:10.1186/1746-4811-9-22)
Supplement: Additional file 1 — Bench protocol for MBP-HAE expression and staining. [file 1746-4811-9-22-S1.docx]

**Protocol for Pro-Q Diamond staining of recombinant autophosphorylating protein kinases in boiled *E. coli* lysate.**

*All conditions in steps 1-4 must be optimized for expression strain, construct, etc. The conditions listed below are only provided as a reference. Molecular Cloning was used as a guide in determining optimum HAE expression conditions.

*All steps shaking at >300 RPM. Aeration of the cultures was found to be a major determinant of recombinant HAE expression levels.

1) Grow culture of strain harboring expression construct overnight at 37 °C in LB media containing the specific antibiotic.

2) The next morning, after the culture has reached stationary phase, inoculate 35 µl into 1 ml of LB + antibiotic (scale to whatever volume is appropriate for your experiment) and grow for roughly 3 hours at 37 °C.

3) Monitor OD_600_ of the cultures until they reach the exponential growth phase between 0.6 and 0.8.

4) Add IPTG to a final concentration of 0.1 mM and grow for 4 hours at 25 °C.

5) Transfer 100 µl of the culture to a 1.5 ml tube and spin at full speed for 1 minute to pellet bacteria. Consider harvesting multiple samples from the same culture as a backup.

6) Remove supernatant with a pipette and either flash freeze in N_2_ and store at -80 °C, or proceed to next step.

7) Put the tube containing the pellet on ice, and add 100 µl 1x SDS sample buffer and resuspend the pellet by pipetting.

8) Boil for 3 minutes to denature the proteins.

9) Centrifuge at full speed for 1 minute to pellet cell debris.

10) Place on ice until ready to load the gel.

11) Allow to warm to room temp just before loading the gel.

12) Load 5 µl into a polyacrylamide gel of appropriate percentage for your protein (4% stacking and 8% separating gel for ~80 kDa MBP-HAE).

13) Run at 80 V until dye front reaches separating gel, then 130 V, for ~ 1.5 hours.

**All subsequent steps performed on rotary platform at ~25 RPM:**

14) When finished, place gel into inert plastic container (polypropylene is good), and add ~50 ml fixation solution (50% Methanol, 10% Acetic acid).

15) Fix for ~30 minutes, replace fixation solution, and fix overnight (the Thelen protocol says it doesn't matter if you fix overnight, but staining seems a little more consistent in our hands when we do).

16) Next morning, remove fixation solution, and rinse 2x for 15 minutes each time with ~50 ml DI

water.

**Steps 17-19 performed in the dark:**

17) Remove the water and stain for 2 hours with 50 ml 1/3x Pro-Q Diamond (diluted in DI water).

18) Remove the stain and rinse 4x for 30 minutes each time with destaining solution (20% acetonitrile, 50 mM sodium acetate pH 4.0).

19) Remove the destain solution and wash twice for 10 minutes with DI water.

20) Image using imager of choice. We use a BioRad Chemidoc, and it works extremely well (the Image Lab software comes shipped with a Pro-Q Diamond program).

21) After imaging, place gel in ~50 ml Coomassie Blue Silver (0.12% Coomassie G-250, 10% ammonium sulfate, 10% phosphoric acid, 20% methanol) for >1 hour.

22) Remove stain, rinse excess stain away with DI water, add destaining solution (10% methanol, 10% acetic acid) and destain for >1 hour.

23) Remove destain solution and wash with twice for 10 minutes with DI water.

24) Image using imager of choice. We use a BioRad Geldoc, because it also utilizes the Image Lab software and works quite well.

25) Proceed to analysis.

**References:**

Agrawal, Ganesh Kumar, and Jay J. Thelen. "Development of a simplified, economical polyacrylamide gel staining protocol for phosphoproteins."*Proteomics* 5.18 (2005): 4684-4688.

Candiano, Giovanni, *et al.* "Blue silver: a very sensitive colloidal Coomassie G‐250 staining for proteome analysis." *Electrophoresis* 25.9 (2004): 1327-1333.

Sambrook, Joseph, and W. Russell David. *Molecular cloning: a laboratory manual. Vol. 3*. Cold Spring Harbor Laboratory Press, 1989.
