# Supplementary figures and images for: A simple in vitro method to measure autophosphorylation of protein kinases
Source: Plant Methods. 2013 Jun 26;9:22. doi: 10.1186/1746-4811-9-22 (PMC3702502; doi:10.1186/1746-4811-9-22)

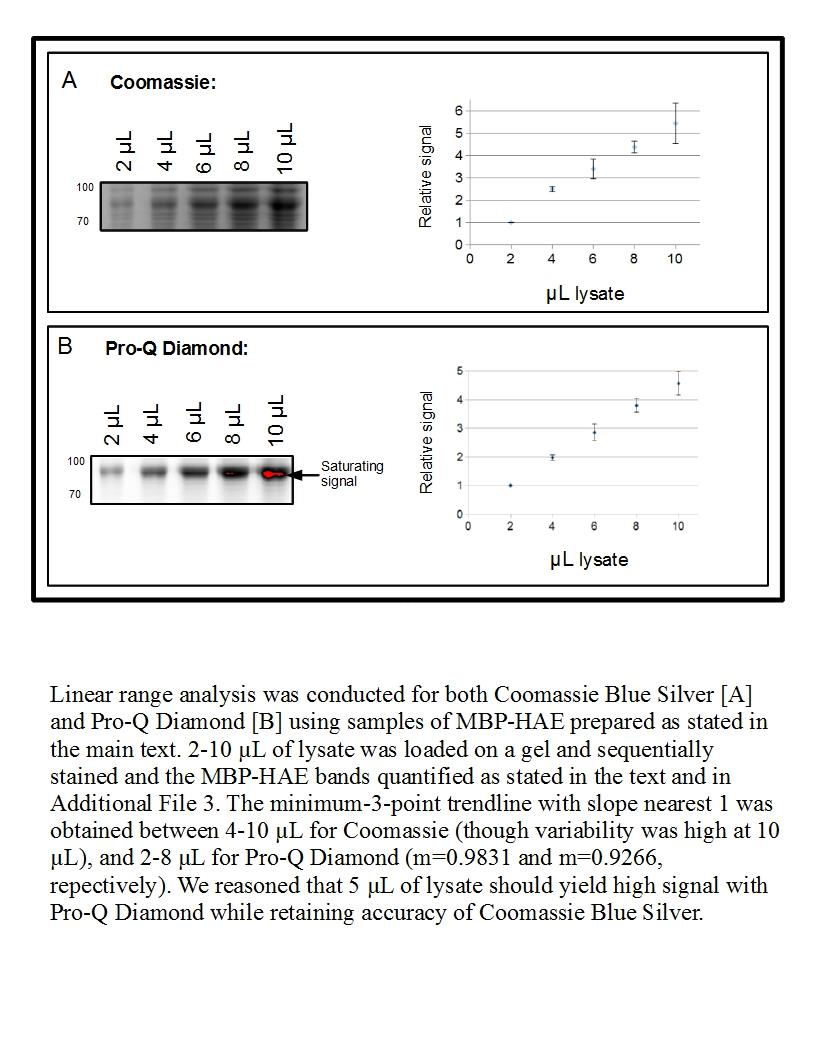

Supplement: Additional file 2 — Linear range analysis of Coomassie Blue Silver and Pro-Q Diamond. [file 1746-4811-9-22-S2.png]

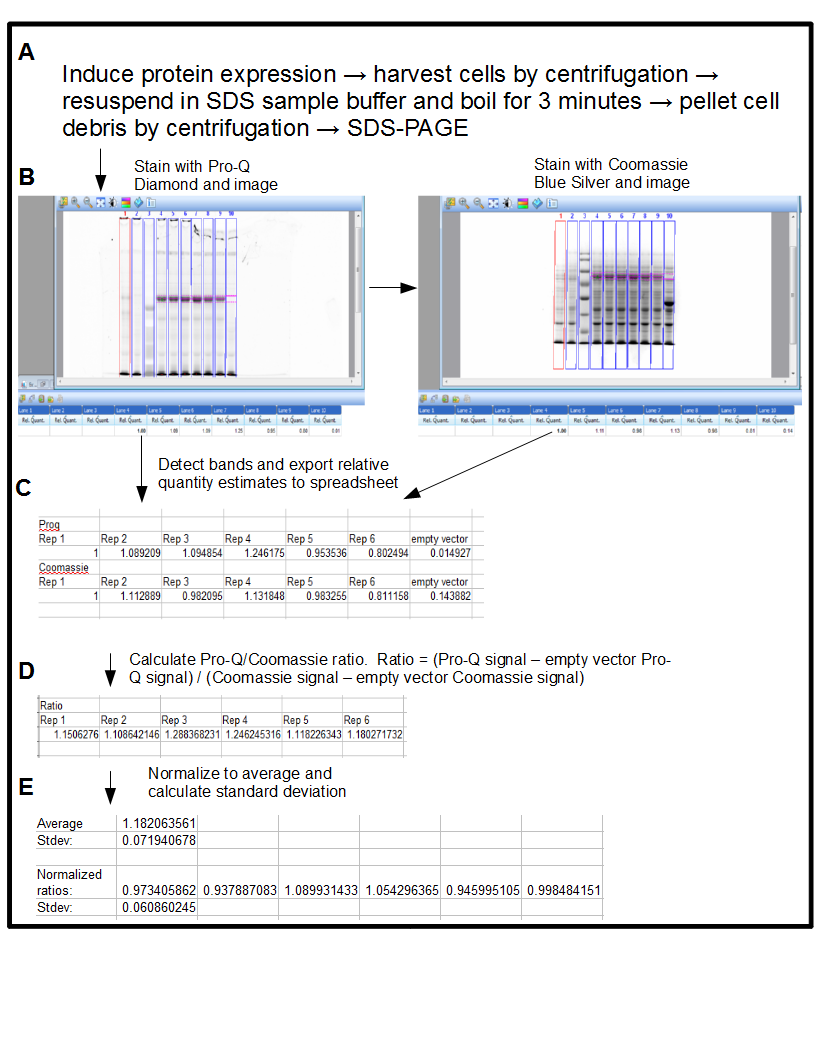

Supplement: Additional file 3 — Diagram of quantification workflow. [file 1746-4811-9-22-S3.png]
